# Histopathological classification of non-functioning pituitary neuroendocrine tumors

**DOI:** 10.1007/s11102-017-0855-1

**Published:** 2017-12-23

**Authors:** Emilija Manojlovic-Gacic, Britt Edén Engström, Olivera Casar-Borota

**Affiliations:** 1 Institute of Pathology, School of Medicine, University of Belgrade, Belgrade, Serbia; 2 Department of Medical Sciences, Endocrinology and Metabolism, Uppsala University Hospital, Uppsala, Sweden; 3 Department of Immunology, Genetics and Pathology, Uppsala University, Uppsala, Sweden; 4 Department of Clinical Pathology, Uppsala University Hospital, Uppsala, Sweden

**Keywords:** Non-functioning pituitary neuroendocrine tumor, Pituitary adenoma, Immunohistochemistry, Transcription factors

## Abstract

Non-functioning pituitary neuroendocrine tumors do not cause endocrine symptoms related to hypersecretion of adenohypophyseal hormones and are clinically characterized by symptoms due to growing sellar tumor mass. Histopathological classification of this tumor group has always been challenging due to their heterogeneity, limited knowledge on their biology, and diverse methodological problems. We have searched PubMed database for data related to the histopathological classification of non-functioning pituitary tumors and methods for its application. Principles of the classification and grading presented in the recently released 4th edition of the World Health Organization classification of endocrine tumors have been summarized. Based on the expression of anterior pituitary hormones and pituitary specific transcription factors, gonadotroph tumors dominate within the group of clinically non-functioning tumors, followed by corticotroph type; however, other less common types of the non-functioning tumors can be identified. Assessment of tumor cell proliferation is important to identify “high-risk adenomas.” A few subtypes of non-functioning tumors belong to the category of potentially aggressive tumors, independent of the cell proliferation rate. Here, we present up to date criteria for the classification of clinically non-functioning pituitary tumors, offer a diagnostic approach for the routine clinical use, and emphasize a need for inclusion of prognostic and predictive markers in the classification.

## Introduction

Non-functioning pituitary adenomas (NFPAs) or, according to a recent proposal [1], non-functioning pituitary neuroendocrine tumors (NF-PitNETs) represent a heterogeneous group of tumors characterized by the lack of endocrine symptoms related to hypersecretion of adenohypophyseal hormones [2]. They are also called “silent” adenomas, emphasizing the lack of endocrine symptoms despite the immunohistochemical expression of anterior pituitary hormones in the tumor cells, in a large majority of the tumors [2]. Even tumors associated with slightly supranormal serum hormone concentrations, but without typical symptoms related to hormone hypersecretion, have been called silent by some authors [3], although the correctness of using the term “silent” in this context may be questionable. Mechanisms behind the silencing of pituitary tumors remain unknown [4].

NF-PitNETs represent more than one-third of PitNETs, with predominance in men and increasing frequency with older age [2, 5, 6]. However, the proportion largely varies in different studies, reaching more than 50% [7], which may, in part, reflect the methodological difficulties in the histopathological diagnostics.

Being clinically silent, NF-PitNETs are usually diagnosed at the stage of macroadenoma, although presentation as an incidentaloma is not uncommon [5, 6]. Around 50% of the tumors infiltrate cavernous sinuses at the time of diagnosis, which limits radical surgery [8, 9]. Even when radiologically radically removed, a significant proportion of the tumors demonstrate regrowth [8, 10, 11]. Mechanisms behind the invasive and/or recurrent growth of NF-PitNETs are largely unknown; currently, there are no good histological predictors of aggressiveness in NF-PitNETs [12, 13].

In contrast to functioning PitNETs, for many of which medical treatment is well-established, pharmacological therapy of NF-PitNETs is still experimental [14], possibly due to the biological heterogeneity of these tumors.

Classification of neuroendocrine pituitary tumors has evolved from that based on the tinctorial features through classification based on immunohistochemical hormone expression and ultrastructural characteristics [15, 16] to the recent World Health Organization (WHO) classification, which is based on the adenohypophyseal cell lineages defined by the expression of adenohypophyseal hormones and transcription factors [2]. With the current immunohistochemical method, only around 2% of PitNETs lack signs of pituitary cell lineage differentiation, being thus classified as null-cell adenomas [17, 18]. Although improvements have been made by including more detailed immunohistochemical characterization of the tumors, omitting majority of the clinically irrelevant ultrastructural subtypes and by adding selected predictive and prognostic markers [2], WHO classification still lacks a reliable correlation between histological parameters and clinical behavior of pituitary tumors, including NF-PitNETs. In recent years, the attempt has been made to combine the histological and immunohistochemical classification with the radiological signs of invasion. Long-term follow-up in more than 400 patients identified pituitary tumors with increased proliferation and MRI- or histologically confirmed invasion as the tumors most prone to recurrence [19, 20].

Classification of NF-PitNETs, which has always been particularly challenging due to the methodological problems limiting their precise characterization, may contribute to: (1) Better understanding of the genesis and biology of this heterogeneous group of pituitary tumors; (2) recognition of factors involved in the invasive and recurrent tumor growth; (3) identification of histological subtypes known for their more aggressive clinical course; (4) definition of predictive markers, which can result in improvement of post-surgical follow-up, better selection of patients suitable for radiation therapy, and hopefully development of new pharmacological therapeutic strategies.

Here, we provide an update on the histopathological classification of NF-PitNETs, present the diagnostic tools required for their classification in routine work, and give an overview of the prognostic and predictive biomarkers.

## Principles of current WHO histopathological classification of NF-PitNETs

The WHO classification recognizes NF-PitNETs as variants of their functioning counterparts, leaving an independent chapter only for null-cell Pit-NETs as the only type without functioning counterpart. The golden standard for the classification of PitNETs is immunohistochemistry with antibodies toward adenohypophyseal hormones, optimally in combination with pituitary-specific transcription factors, in order to define pituitary cell lineage differentiation of the tumor [2].

As for PitNETs, in general, grading of NF-PitNETs is based on three categories, comprising the most frequent “typical adenoma” and exceptional “pituitary carcinoma.” The third term, “atypical adenoma,” recommended by the 2004 WHO classification [15], has been abandoned due to the lack of the ability to predict aggressive behavior of PitNETs [2, 21]. Subsequently, the term “high risk pituitary adenoma” evolved, comprising PitNETs with increased cell proliferation assessed by mitotic count and Ki-67 proliferative index and signs of invasive growth evaluated by MRI and/or histology. Furthermore, the recent WHO classification defines certain subtypes of PitNETs that tend to behave more aggressively. Notably, three out of the five types of potentially aggressive PitNETs behave or may potentially behave as non-functioning: silent corticotroph, poorly differentiated Pit-1 lineage tumor, and sparsely granulated somatotroph tumor [2]. This accentuates the need for correct histological phenotyping of NF-PitNETs, which may be challenging particularly in cases with sparse or no immunohistochemical hormone expression.

Insufficient standardization of immunohistochemical procedure, the lack of reliable and specific antibodies, and problems with the interpretation of immunohistochemical stainings are still making difficulties, both in phenotyping of the tumors, particularly non-functioning ones [2, 22], and in the assessment of proliferation [2].

Molecular analyses are still not integrated in routine diagnostics of NF-PitNETs since genetic mechanisms of their genesis have not yet been clarified [23]. However, it should be kept in mind that a small proportion of tumors related to both sporadic and germ-line mutations may present as NF-PitNETs [24]. The mutation of ubiquitin-specific protease 8 (USP8), which is frequently found in corticotroph tumors associated with Cushing disease, is not observed in silent corticotroph tumors, representing a rare example of molecular genetic differences between silent and secreting variants within the same group of pituitary tumors [25]. Sporadic gain-of-function mutations of the *GNAS* gene coding for the Gsα protein occur in approximately 40% of somatotroph tumors causing acromegaly [23, 24]; however, there are no systemic data on the presence of the *GNAS* mutations in silent somatotroph tumors. Germ-line mutations are usually associated with hormone producing PitNETs [23, 24]. In multiple endocrine neoplasia syndrome type 1 (MEN1), though, prolactinomas and non-functioning pituitary tumors are almost equally represented [24]. Another group of syndromic disorders in which non-functioning PitNETs can occur is related to mutations in the succinate dehydrogenase genes (*SDHx*). The *SDHx* mutations-associated PitNETs have been reported to demonstrate characteristic histopathological appearance with vacuolar change in the tumor cells [26].

## Pituitary lineage specific classification of NF-PitNETs

NF-PitNETs are divided into eight subtypes, according to the WHO 2017 classification (Table 1), based on the immunohistochemical expression of adenohypophyseal hormones and pituitary-specific transcription factors [2].

**Table 1:** Histopathological types of NF-PitNETs with diagnostic and potential prognostic/predictive immunohistochemical markers

NF-PitNET type	Transcriptional factor	Hormone staining	LMWCK	Prognostic/predictive markers
Gonadotroph	SF-1 (GATA-2, ERα)	β-FSH, β-LH, α-SU	Variable	ERα, SSTRs
Corticotroph^a^	T-Pit	ACTH	Diffuse	
Type 1 (densely granulated)	T-Pit	Diffuse, strong ACTH	Diffuse	
Type 2 (sparsely granulated)	T-Pit	Weak, patchy ACTH	Diffuse	
Crooke-cell	T-Pit	Periphery of the cell	Ring-like	
Somatotroph	Pit-1	GH		SSTRs
Sparsely granulated^a^	Pit-1	Diffuse, strong	Fibrous body	
Densely granulated	Pit-1	Weak, patchy	Diffuse	
Thyrotroph	Pit-1 (GATA-2)	TSH, α-SU	Variable	SSTRs
Lactotroph	Pit-1 (ERα)	PRL	Variable	
Sparsely granulated	Pit-1 (ERα)	Perinuclear, Golgi zone PRL	Variable	
Densely granulated	Pit-1 (ERα)	Diffuse PRL	Variable	
Acidophilic stem cell adenoma	Pit-1 (ERα)	Focal and variable PRL, GH	Fibrous body (inconsistent)	
Plurihormonal Pit-1^a^	Pit-1	GH, PRL, TSH, α-SU	Variable	
Null-cell	None	None	Variable	
Double/triple NF-PitNET	More than one	Variable	Variable	

Increased proliferation and MRI-confirmed invasion are criteria for high-risk adenoma in all types. MGMT is a potential predictive marker for response to temozolomide in aggressive PitNETs of all types

^a^NF-PitNET types with potential aggressive biological behavior

The use of antibodies toward anterior pituitary hormones: growth hormone (GH), prolactin (PRL), follicle-stimulating (FSH), luteinizing (LH), thyroid stimulating hormone (TSH), and adrenocorticotroph hormone (ACTH) is required for phenotyping of PitNETs and for recognition of potentially aggressive subtypes. Antibody toward α-subunit of glycoprotein hormones (TSH, FSH, LH) may be useful in cases with sparse hormone expression [2, 15].

**Fig. 1:**
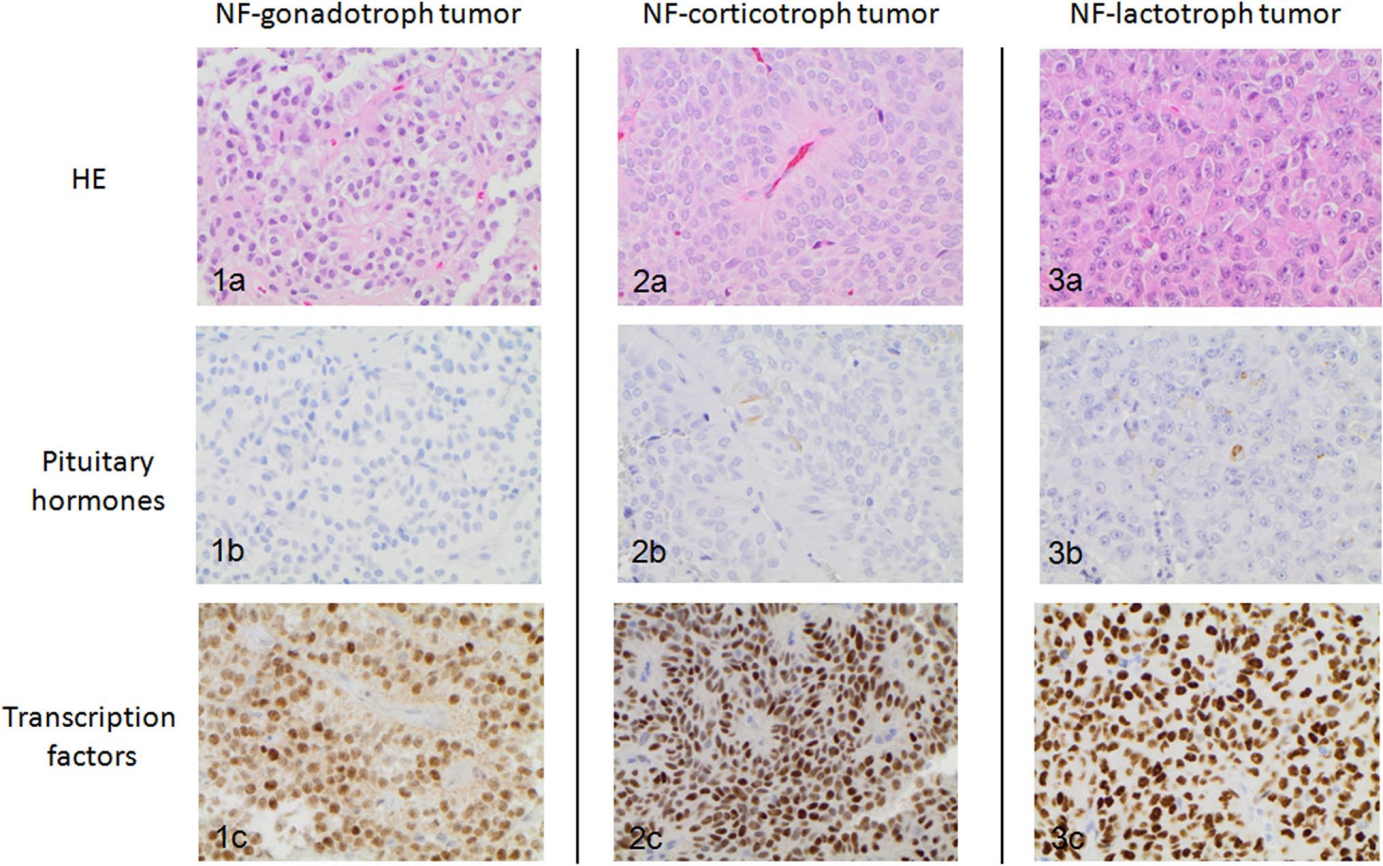
Importance of pituitary transcription factors in PitNETs with sparse or no hormone-immunolabeling: NF-gonadotroph tumor** 1a** hematoxylin eosin staining** 1b** negative immunolabeling for gonadotroph hormones (FSH and LH)** 1c** nuclear expression of SF-1; NF-corticotroph tumor** 2a** hematoxylin eosin staining** 2b** sparse ACTH expression** 2c** nuclear expression of T-Pit; NF-lactotroph tumor** 3a** hematoxylin eosin staining** 3b** sparse expression of prolactin** 3c** nuclear expression of Pit-1 (Magnification x400)

Differentiation of the three main cell lineages in adenohypophysis is mediated by transcription factors [27], which are also retained in the tumors, both silent and functional, showing differentiation toward the adenohypophysial cells. Three of the pituitary specific transcription factors are recommended in routine diagnostics: pituitary transcription factor 1 (Pit-1), steroidogenic factor 1 (SF-1), and T-box family member TBX19 (T-Pit) [2, 22] (Fig. 1).

Pit-1 plays a role in the differentiation of somatotroph, lactotroph, and thyrotroph cells and respective tumors [28], including the plurihormonal variants [29]. Good-quality Pit-1 antibodies for immunohistochemistry are available.

SF-1 acts as transcription factor for differentiation of gonadotroph cells. Subsequently, it is expressed in gonadotroph PitNETs, both in silent and rare clinically functioning tumors [30]. Monoclonal anti-SF-1 antibody (clone N1665) has usually been used, albeit with inconsistent results.

T-Pit is required for the transcription of proopiomelanocortin (POMC), the precursor polypeptide to ACTH [31, 32], being a marker of corticotroph differentiation. A novel purified polyclonal antibody toward T-Pit has recently been published and is expected to be available soon [18].

Although a complete antibody panel will still be required for precise classification and for characterization of some unusual types of PitNETs, some authors propose the use of transcription factors as the first line of immunohistochemical screening of PitNETs, especially in the laboratories with high workload [22].

Although not used routinely, estrogen receptor α (ERα) and guanine–adenine–thymine–adenine binding protein 2 (GATA-2) are also recognized as transcription factors involved in the differentiation of gonadothroph, lactotroph, and thyrotrops cells, and their respective tumors [2, 27, 33, 34].

### Subtypes of NF-PitNETs

*Non-functioning/silent gonadotroph tumors* are SF-1 cell lineage derived tumors that typically demonstrate at least focal immunolabeling for β-FSH, β-LH, and α-subunit [2]. Despite the methodological difficulties related to suboptimal quality of the available anti SF-1 antibodies, the nuclear labeling can usually be detected in a significant proportion of tumor cells, enabling the diagnosis in cases with sparse or no gonadotroph hormone expression [22]. Gonadotroph tumors comprise almost 80% of NF-PitNETs, when both antibodies toward gonadotroph hormones and SF-1 are used in classification [18, 35]. However, they have been underestimated and usually classified as null cell adenomas in previous studies, in which transcription factors were not available [36, 37]. It is the only type of PitNET where the non-functioning form dominates [38, 39].

*Non-functioning/silent corticotroph tumors* are T-Pit lineage related tumors, usually with sparse ACTH expression without clinical evidence of Cushing disease [2, 40, 41]. They constitute about 15% of NF-PitNETs, thus, representing the second largest group of these tumors [18, 39]. The proportion of silent corticotroph tumors among NF-PitNETs is expected to increase with greater use of immunohistochemistry with anti-T-Pit antibody allowing for identification among the tumors with sparse or no ACTH expression [18]. Similar to their functioning counterparts, silent corticotroph tumors can be morphologically and ultrastructurally subdivided into densely and sparsely granulated [2, 42]. Rarely, Crooke-cell adenoma with a typical perinuclear ring-like accumulation of cytokeratin and relocation of ACTH positivity to the sub-membranous zone can manifest as clinically silent [41, 43]. Transformation of silent corticotroph tumors into functioning PitNET (or, exceptionally, vice versa) has been comprehensively analyzed in corticotroph tumors [40, 44–46]. However, the mechanisms remain unclear despite several potential explanations [40, 47–49]. Independently of their morphological variants, silent corticotroph tumors are recognized as tumors with the more aggressive clinical behavior due to their tendency for invasive growth, apoplexy, and recurrences [2, 40, 50, 51].

*Non-functioning/silent somatotroph tumors* are Pit-1 and GH immunoreactive tumors without clinical signs of acromegaly [2, 52, 53]. They represent 2–3% of all pituitary tumors [53]. Similar to their much more frequent functioning counterparts, they can be divided into sparsely and densely granulated somatotroph tumors based on low molecular weight cytokeratin (LMWCK) [CK7/8 (Cam5.2) and CK18] staining, demonstrating either fibrous bodies or diffuse cytoplasmic pattern (Table 1) [2, 53]. NF-somatotroph PitNETs are predominantly sparsely granulated, in contrast to functioning somatotroph PitNETs, where the frequency of sparsely and densely granulated tumors is equal [52, 53] or in favor of densely granulated subtype [54, 55]. They have a lower proportion of GH-immunoreactive cells, which suggests lower differentiation. Majority of silent somatotroph tumors co-express prolactin [53]. Sparsely granulated somatotroph tumors are, according to the WHO classification [2], designated as more aggressive tumors based on several studies demonstrating that they are usually larger and more invasive compared to the densely granulated subtype [54–56]. In patients with acromegaly, the sparsely granulated somatotroph tumors are less responsive to somatostatin analogues, probably due to the lower expression of somatostatin receptors (SSTRs) [55].

*Non-functioning/silent lactotroph PitNET* are PRL-immunoreactive tumors with no clinical signs of hyperprolactinemia, apart from elevated prolactin levels due to the stalk effect [57]. In addition to transcription factor Pit-1, they also express estrogen receptor alpha [2]. Pure silent lactotroph tumors are exceptional. More often, prolactin is co-expressed with GH in Pit-1 positive tumors. Silent lactotroph tumors are very rare in surgical, but not in autopsy series [37, 58]. Both silent and functioning lactotroph tumors are sub-classified into sparsely granulated, demonstrating Golgi-like prolactin immunoreactivity and densely granulated with diffuse cytoplasmic expression of prolactin in the tumor cells [2]. Acidophil stem-cell adenoma has only been exceptionally described in silent form [37]. More aggressive behavior of the functioning lactotroph tumors in men seems to correlate with the lower expression of ERα compared with the lactotroph tumors in women [33]. However, the expression of ERα and its correlation with the clinical characteristics of silent lactotroph tumors is still obscure. Rare silent lactotroph tumors is important to identify, as addition of dopamine agonists may be considered in the treatment, although the effects are less well documented than in the clinically-functioning prolactinomas [59].

*Non-functioning/silent thyrotroph tumors* belong to Pit-1 lineage tumors and express β-TSH without clinical and biochemical signs of central hyperthyroidism [2, 60, 61]. They seem to occur with slightly higher frequency than their rare functioning counterparts [62, 63]. Silent and functioning thyrotroph tumors behave in a similar manner regarding the recurrence rate and time from surgery to the recurrence [62]. Non-functioning thyrotroph tumors rarely change its endocrine activity, transforming into functioning thyrotroph PitNET [35, 64].

*Null-cell PitNETs* are defined by the lack of immunohistochemical evidence of differentiation toward any anterior pituitary cell, using antibodies to adenohypophysial hormones and transcription factors [2]. They comprise only a few percent of all pituitary tumors [18]. However, their frequency has frequently been overestimated due to suboptimal immunohistochemical protocols and lack of reliable antibodies to pituitary-specific transcription factors. Further investigations are needed to elucidate whether null-cell adenomas really exist, or whether this category just reflects methodological limitations in the current diagnostic procedure. Since null-cell adenomas are the diagnosis of exclusion, they may sometimes pose the challenge in differential diagnosis with other, non-adenohypophysial neuroendocrine tumors of the sellar region [65].

*Plurihormonal poorly differentiated Pit-1 positive tumors* (previously designated as “silent subtype 3 adenoma”) are rare tumors composed of large polygonal or spindle shaped cells, with atypical nuclei, sometimes containing inclusions, so called nuclear spheridia that can be observed on routine HE stains or, more readily, on electron microscopy [29, 66–68]. They may demonstrate variable and patchy immunoreactivity for GH, PRL, and TSH in different combinations. Although traditionally categorized as silent, plurihormonal Pit-1 positive tumors are associated with clinical signs of hypersecretion of Pit-1 lineage hormones in about 30% of the cases [29]. The correct diagnosis of this uncommon type of NF-PitNETs is important since it belongs to the group of potentially aggressive tumors, usually macroadenomas with propensity to invade cavernous sinus and clivus and to recur [2, 29, 66, 68].

*Double/triple pituitary tumors* are very rare tumors composed of demarcated components originating from different cell lineages, which can be confirmed by expression of more than one pituitary specific transcription factors. In the same tumor, silent and hormone-secreting components may be present [2]. Based on sporadic reports and small series, double/triple pituitary tumors seem to be clinically active in the majority of cases [69]. Combination of two or three NF PitNETs has been more frequently reported in autopsy material, suggesting that some tumors have been clinically silent [58, 70].

## Prognostic markers of NF-PitNETs

### General prognostic markers for grading of NF-PitNETs: mitotic count, Ki-67 index, p53

#### Mitotic count

Cell proliferation is an important prognostic marker in neuroendocrine tumors in general [71]. The prognostic significance of mitotic count in pituitary neuroendocrine tumors cannot be overestimated [19, 20]. Thorough assessment of the mitoses throughout the surgical specimen is highly recommended [2, 19, 20], with special focus on the areas with hot-spot Ki-67 immunoreactivity, when present [2].

#### Ki-67 proliferative index

The Ki-67 assessment by using immunohistochemistry usually with monoclonal MIB1 antibody is mandatory for the assessment of proliferation. It seems that Ki-67 does not differ significantly between hormone-secreting and non-secreting pituitary tumors [72]. Methodological problems, interpretational difficulties, and use of different cut-off values in different studies [73, 74] may explain why the interpretation of Ki-67 has recently been reformulated, moving from the cut-off of 3% in the previous WHO classification of pituitary tumors [15] to the estimation of the percentage of Ki-67 positive nuclei without precise cut-off in the 2017 WHO classification [2]. Studies on NF-PitNETs revealed that proliferative Ki-67 index remains the second parameter in the prediction of recurrence, after invasion of surrounding structures [75].

Proliferative Ki-67 index and mitotic activity should be estimated carefully in apoplectic PitNETs, since proliferation of inflammatory cells and cells surrounding necrosis should not be interpreted as proliferation of tumor cells. In this situation, proliferation should be evaluated in the well-preserved tumor areas, if possible, and use of additional immunolabeling with a lymphocytic marker should be considered to estimate the proportion of inflammatory cells among the Ki-67 reactive cells.

#### Tumor suppressor gene p53 as a marker of proliferation in PitNETs

Even though mutation of p53 is very rarely detected in sporadic PitNETs [76], its nuclear accumulation detected by immunohistochemistry suggested potential aggressive behavior of pituitary tumors in previous studies [77], which resulted in inclusion of p53 among the criteria for the classification of “atypical adenoma” in the previous WHO classification [15]. Nevertheless, there is still no clear consensus about its interpretation in pituitary tumors, including non-functioning ones [78, 79].

Bearing in mind that none of the above markers of proliferation has been proved to be prognostic independently, their combination was verified to be prognostic, particularly when associated with invasive tumor growth [19, 20].

#### Additional potential prognostic markers in NF-PitNETs: ERα, E-cadherin, MGMT

Correlation between ERα and invasiveness in NF-PitNETs has been reported in a previous study [80]. Recently, absence of ERα was found to correlate with the rate of postoperative radiotherapy or surgical re-intervention in men with NF-PitNETs [35].

Role of E-cadherin, a marker of epithelial differentiation, in the invasiveness of NF-PitNETs is still unclear, despite an earlier study that could not demonstrate the correlation between E-cadherin expression and cavernous sinus invasion of NF-PitNETs [36].

Prognostic value of *O*-6-methylguanine-DNA methyltransferase (MGMT) is still controversial. In a recent meta-analysis, lower immunohistochemical expression of MGMT was associated with recurrence of PitNETs regardless of the functional status of the tumors [81].

More studies are needed before these additional prognostic markers may find their place in routine practice and eventually in the classification of NF-PitNETs.

### Specific prognostic categories of NF-PitNETs

WHO classification recognizes five categories of pituitary adenomas that are shown to be more clinically aggressive regardless of their histological grading: sparsely granulated somatotroph adenomas, lactotroph macroadenomas in men, Crooke cell adenoma, silent corticotroph adenomas, and plurihormonal Pit-1 positive adenoma [2]. One of them, silent corticotroph tumor, behaves as clinically non-functioning and two of them, plurihormonal Pit-1 lineage tumor and sparsely granulated somatotroph tumor, may potentially be clinically silent.

### Non-functioning pituitary carcinomas

Metastasizing Pit-NETs or pituitary carcinomas are exceptional, and majority of them are hormone-secreting. However, non-functioning pituitary carcinomas of gonadotroph, corticotroph, or null-cell type have occasionally been reported. NF-pituitary carcinomas may also be underdiagnosed as their metastases can remain asymptomatic for years [82, 83].

## Potential predictive markers (somatostatin receptors, dopamine receptor, MGMT, MSH)

As no medical treatment is available so far for patients with NF-PitNETs, recognition of markers that potentially could predict a response to established or novel therapies is much needed.

SSTRs, particularly type 3 and to lesser degree types 2 and 5, are expressed in gonadotroph tumors and may thus represent potential target of multireceptor somatostatin analogues such as pasireotide in patients with aggressive NF-PitNETs [35, 84, 85].

Dopamine receptors have been demonstrated on mRNA level in NF-PitNETs [86]. In a recent study, no correlation was found between DR2 protein and mRNA expression and response to treatment in patients with NF-PitNETs who received dopamine agonists aiming to prevent tumor growth after the surgery [87]. Immunohistochemical studies on DRs are unfortunately few due to the methodological difficulties related to anti-DR-antibodies.

Lower MGMT expression assessed by immunohistochemistry was found to correlate with better response to temozolomide in a study of 24 patients with aggressive PitNETs and pituitary carcinoma, of which five had aggressive NF-PitNET [88]. However, the results regarding MGMT as a predictor of temozolomide effect in pituitary tumors are still contradictory [89, 90].

DNA mismatch protein MSH6 expression in NF-PitNETs has been suggested as a predictor of temozolomide effect in PitNETs [91]; however, other studies did not confirm that [88].

## Methodological limitations in the histopathological diagnostics of NF-PitNETs

Immunohistochemistry is essential in the classification of pituitary tumors. It is a widely-used method in pathology because it can be highly sensitive and specific in protein detection on tissue slides, fast, relatively inexpensive, and can be automated. At the same time, different preanalytical conditions such as ischemic time and duration of fixation are difficult to control even in the standardized clinical laboratory settings. Immunohistochemical protocols require continuous optimization through internal and external validation; read-out of the immunohistochemical results is human-dependent and prone to subjective interpretation [92].

In the context of pituitary tumor pathology, the preanalytical factors can potentially affect the expression of adenohypophysial hormones. The combined use of antibodies toward the adenohypophysial hormone and transcription factors, at least in tumors with sparse hormone expression, can increase the accuracy in the phenotyping of PitNETs. However, experiences with the T-Pit antibody are limited in routine praxis as it has only recently been available, and the most often used anti SF-1 antibody, clone N1665, produces inconsistent results even in the laboratories with expertise within pituitary pathology. When it comes to the proliferative marker Ki-67, disagreement about the quantification method and a lack of the assessment recommendations are additional problems. Experience with the digital quantification of Ki-67 in clinical praxis is limited, but should be considered as a tool to reduce the intra- and inter-observer variations in the assessment [75].

## Conclusion

Non-functioning pituitary tumors represent a heterogeneous group of PitNETs, whose precise histopathological classification depends upon detailed immunohistochemical evaluation of adenohypophysial hormone expression. Assessment of mitotic account, proliferative Ki-67 index, and tumor invasiveness is important to identify potentially aggressive tumors. Application of pituitary specific transcription factors plays an important role in recognition of less differentiated tumor types, some of which may demonstrate aggressive behavior. Predictive markers should be included in the classification, particularly when pharmacological therapy is under consideration. Further investigations are needed to recognize additional prognostic and predictive markers in clinically NF-PitNETs.
